# Insulin receptor compensates for IGF1R inhibition and directly induces mitogenic activity in prostate cancer cells

**DOI:** 10.1530/EC-13-0086

**Published:** 2014-02-28

**Authors:** Doron Weinstein, Rive Sarfstein, Zvi Laron, Haim Werner

**Affiliations:** 1 Department of Human Molecular Genetics and BiochemistrySackler School of Medicine, Tel Aviv UniversityTel Aviv, 69978Israel; 2 Endocrinology and Diabetes Research UnitSchneider Children's Medical CenterPetah Tikva, 49202Israel

**Keywords:** insulin, insulin receptor, insulin-like growth factor-1 (IGF1), IGF1 receptor, prostate cancer

## Abstract

Hyperinsulinemia is a major complication associated with the development of insulin resistance. In addition to its normal spectrum of metabolic effects, insulin can act as a growth factor and has the ability to promote mitogenic activity. Thus, hyperinsulinemia is regarded as a potentially important cancer risk factor among diabetic patients. However, the mechanisms of action of insulin in the specific context of prostate cancer (PCa) and, in particular, the specific receptor that mediates its actions have not been elucidated yet. The aims of this study were to investigate whether insulin can directly induce mitogenic activities in PCa-derived cell lines and to examine the mechanisms responsible for these actions. To this end, we used several PCa-derived cell lines, representing early and advanced stages of the disease. Our results indicated that insulin induces cell proliferation in a dose-dependent fashion in the LNCaP, C4-2, and P69 cell lines. We also demonstrated that insulin enabled LNCaP and C4-2 cells to progress through the cell cycle. Immunoprecipitation assays revealed that insulin activated the insulin receptor (INSR), but not the IGF1 receptor (IGF1R). In addition, INSR was able to compensate for and mediate IGF1 mitogenic signals following IGF1R inhibition. In conclusion, insulin exhibits direct mitogenic activities in PCa cells, which are mediated exclusively through the INSR. Further research is needed to fully dissect the molecular mechanisms underlying the biological actions of insulin in PCa.

## Introduction

Prostate cancer (PCa) is the second most frequently diagnosed cancer in men and the third most common cause of death in men aged over 50 years in developed countries. Apart from age, race, and a positive family history are among the strongest known risk factors for the disease (1). The incidence of PCa has substantially increased in the past two decades, making it one of the most severe public health threats in current medicine. PCa, if diagnosed during its early stages, can be cured surgically or by radiotherapy. However, few therapeutic choices are available for patients with metastatic disease and those with castration-resistant PCa (2). Therefore, developing new treatment options to treat these aggressive forms of PCa has a very high priority. Novel agents targeting pathways involved in proliferation, apoptosis, or immune modulation have entered clinical trials (3). For example, epidermal growth factor receptor tyrosine kinase and vascular endothelial growth factor inhibitors are effectively used in clinical settings (2, 4).

The insulin-like growth factor (IGF) system has an important role in the normal growth and development of the prostate gland (5). In addition to its physiological role, epidemiological, clinical, and experimental evidence suggests an association between IGF system components and PCa development (1). In particular, a close functional connection has been identified between the IGF1 receptor (IGF1R), a transmembrane heterotetramer primarily involved in the mediation of IGF1 signals, and the androgen receptor (AR), a key element in prostate gland function. In recent years, the IGF1R has emerged as a promising therapeutic target in cancers, including prostate tumors.

The clinical and metabolic importance of insulin has been well established (6, 7). In addition to its key role in the maintenance of glucose homeostasis (8), insulin and its receptor (INSR) are involved in the development of a number of metabolic conditions, including obesity and type 2 diabetes (9). The fact that these diseases are regarded as risk factors for cancer raises the question whether there is a direct connection between insulin and INSR and cancer. Elevated levels of insulin and INSR have been reported in several human cancers, including breast, colon, and lung, suggesting the existence of a common etiological link. However, the mechanisms of action of insulin in the specific context of PCa and, in particular, the specific receptor that mediates its actions have not been elucidated yet.

The INSR shares a high structural homology with the IGF1R (84% similarity in the tyrosine kinase domain, 45–65% in the ligand-binding domain, and more than 50% in the overall amino acid sequence). In addition, ligand-dependent activation of the INSR and IGF1R activates almost identical downstream signaling cascades (10). However, whereas the IGF1R has been identified as a potential therapeutic target in cancer, such a validation is still lacking for the closely related INSR. The aims of the present study were to examine the hypothesis that insulin can directly induce mitogenic activity in PCa cells via its cognate receptor and to investigate the ability of INSR to compensate for and mediate IGF1 mitogenic signals following IGF1R inhibition.

## Subjects and methods

### Cell lines and treatments

The P69 and M12 PCa cell lines were a gift from Dr Joy L Ware (Medical College of Virginia, Richmond, VA, USA). The derivation of these cell lines has been described by Bae *et al*. (11). Briefly, the P69 cell line was derived by immortalization of human primary prostate epithelial cells with SV40 large T antigen. P69 cells are responsive to IGF1 and are rarely tumorigenic. The M12 cell line was derived from the P69 cell line by selection for tumor formation in nude mice. M12 cells are tumorigenic and highly metastatic and exhibit reduced IGF1 responsiveness. P69 and M12 cells were maintained in RPMI 1640 medium (Biological Industries, Kibbutz Beit Haemek, Israel) containing 10% FBS, 2 mM glutamine, and 50 μg/ml gentamicin sulfate. The PC3, LNCaP, and C4-2 cell lines were obtained from the American Type Culture Collection (Manassas, VA, USA). Frozen ampoules were thawed and used for up to 3 months (∼20 passages). The PC3 PCa-derived cell line was established from metastatic tumor tissue from a lumbar vertebra of a 62-year-old Caucasian man (12). The LNCaP cell line was established from a lymph node of a 50-year-old Caucasian man with a confirmed diagnosis of metastatic prostate carcinoma (13). C4-2 is an androgen-independent, androgen-insensitive subline of LNCaP cells. PC3, LNCaP, and C4-2 cells were maintained in RPMI 1640 medium supplemented with 10% FBS, glutamine, and gentamicin sulfate. The cells were treated with IGF1 (PeproTech Ltd., Rocky Hill, NJ, USA) or regular insulin (Biological Industries). In some experiments, the cells were treated with IGF1, separately or in combination with either 10 mg/ml cixutumumab (IMC-A12, a fully human antibody antagonist to the human IGF1R (ImClone Systems, New York, NY, USA)) or 1–10 mM tyrphostin AG1024, a selective IGF1R inhibitor (Calbiochem, Darmstadt, Germany), for 48 h.

### Western immunoblotting

The cells were serum-starved overnight and then incubated with IGF1 or insulin for the indicated time periods. After incubation, the cells were harvested and lysed in a buffer containing protease inhibitors (9803, Cell Signaling Technology, Inc., Beverly, MA, USA). Protein content was determined using the Bradford reagent (Bio-Rad) and BSA as a standard. The samples were electrophoresed through 10% SDS–PAGE, followed by blotting of the proteins onto nitrocellulose membranes. After blocking with 5% skimmed milk or 3% BSA, the blots were incubated overnight with the antibodies listed below, washed, and incubated with the appropriate HRP-conjugated secondary antibody. Antibodies against IGF1R (IGF1R β-subunit (C-20)), ERK1 (K-23), and AR (441) were purchased from Santa Cruz Biotechnology, Inc. An antibody against tubulin (B-5-1-2) was purchased from Sigma–Aldrich Co. Antibodies against phospho-IGF1R/INSR (3024), IGF1R β-subunit (3027), INSR β-subunit (3025), phospho-AKT (9271), AKT (9272), and phospho-ERK1/2 (9106) were obtained from Cell Signaling Technology, Inc. The secondary antibodies were HRP-conjugated goat anti-rabbit IgG (1:50 000) and donkey anti-mouse IgG (1:25 000; Jackson ImmunoResearch Laboratories, West Grove, PA, USA). Proteins were detected using the SuperSignal West PicoChemiluminescent Substrate (Pierce, Rockford, IL, USA). Tubulin was used as a loading control for total proteins.

### Immunoprecipitation assays

Cell lysates (70 μg) were immunoprecipitated by incubation with anti-IGF1R β-subunit (1:40) or anti-INSR (1:50) overnight at 4 °C. Protein A/G agarose beads (SC-20003; Santa Cruz Biotechnology, Inc.) were added to the samples and incubated for 2 h. The samples were then washed with PBS, mixed with sample buffer, boiled for 5 min at 95 °C, and electrophoresed through 10% SDS–PAGE. Finally, the membranes were blotted as described above with anti-phospho-IGF1R/INSR, anti-IGF1R, or anti-INSR.

### Transfections and luciferase assays

For transient transfection experiments, an *INSR* promoter–luciferase reporter construct was employed. The *INSR* promoter construct, subcloned in the pGL3 vector (a gift from Dr Antonio Brunetti, University Magna Graecia, Catanzaro, Italy), includes the region from nucleotides −2 to −1823 upstream of the translation initiation site (14). Stable C4-2 and PC3 cells transfected with a WT-AR expression vector (or empty pcDNA3 vector) were seeded in six-well plates and transfected with 1 μg of the *INSR* promoter reporter along with 0.2 μg of a β-galactosidase plasmid (pCMVβ; Clontech), using the jetPEI transfection reagent (Polyplus, Illkirch, France). Control transfections included equal amounts of DNA using the corresponding empty vectors. The WT-AR vector was provided by Dr Norman Greenberg (Fred Hutchinson Cancer Research Center, Seattle, USA). Dihydrotestosterone (DHT) was purchased from Sigma–Aldrich Co. and added at a concentration of 10^−8^ M during the last 24 h of the incubation period. The cells were harvested 48 h after transfection and luciferase and β-galactosidase activities were measured. Promoter activities are expressed as luciferase values normalized to β-galactosidase activity.

### Proliferation assays

LNCaP, C4-2, and PC3 cells were seeded in six-well plates (5×10^4^ LNCaP cells/well, 2×10^4^ C4-2 cells/well, and 3×10^4^ PC3 cells/well). After 24 h, the cells were washed with PBS, and the medium was replaced with a starvation medium (RPMI 1640 without FBS). The cells were then treated with 0–500 ng/ml insulin and, after 48 h, harvested, stained with 0.2% Trypan blue, and counted using a hemocytometer. The cell proliferation rates of P69 and M12 cells, and those of LNCaP and C4-2 cells in compensation experiments, were assessed using the methyl thiazolyl blue tetrazolium bromide (MTT) method (15). Briefly, the cells were seeded in 24-well plates in triplicate. After 24 h, the cells were treated with insulin or IGF1, in the presence or absence of A12 for 48 h, after which cell viability was assessed. The color developed was quantitated by measuring absorbance at a wavelength of 530 nm on a microplate reader (SpectraMax 190, Molecular Devices, Sunnyvale, CA, USA). Cell viability is expressed as a percentage of optical density values obtained upon treatment relative to controls.

### Cell-cycle analysis

The cells were seeded in six-well plates (5×10^4^ LNCaP cells/well, 2×10^4^ C4-2 cells/well, and 3×10^4^ PC3 cells). After 24 h, the cells were washed with ice-cold PBS and then serum-starved for an additional 24 h. The cells were then incubated in the presence or absence of insulin (0–500 ng/ml) for 48 h. After incubation, the cells were washed with PBS, trypsinized, permeabilized with Triton X-100 (4%), and stained with propidium iodide (50 mg/ml). Stained cells were analyzed using a FACSort flow cytometer (Becton Dickinson, Franklin Lakes, NJ, USA).

## Results

### Analysis of basal IGF1R and INSR levels in PCa cell lines

**Figure 1:**
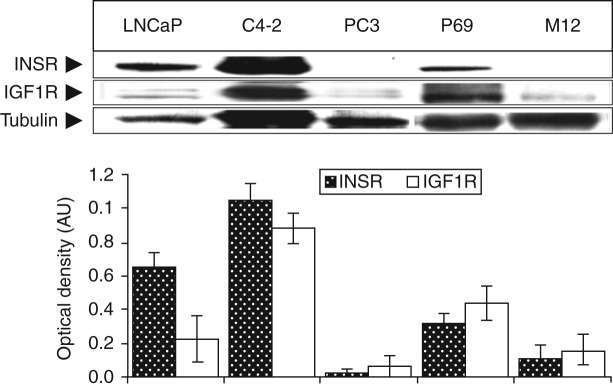
(Top panel) Western blot analysis of IGF1R and INSR levels in PCa cell lines. Cells were lysed and extracts (70 μg) were electrophoresed through SDS–PAGE, followed by transfer and incubation with IGF1R β-subunit and INSR β-subunit antibodies. (Bottom panel) Scanning densitometric analysis of basal IGF1R and INSR levels. Bars represent IGF1R and INSR values (AU, arbitrary units) normalized to the corresponding tubulin levels. Results of a typical experiment, repeated three times with similar results, are shown in the figure.

**Figure 2:**
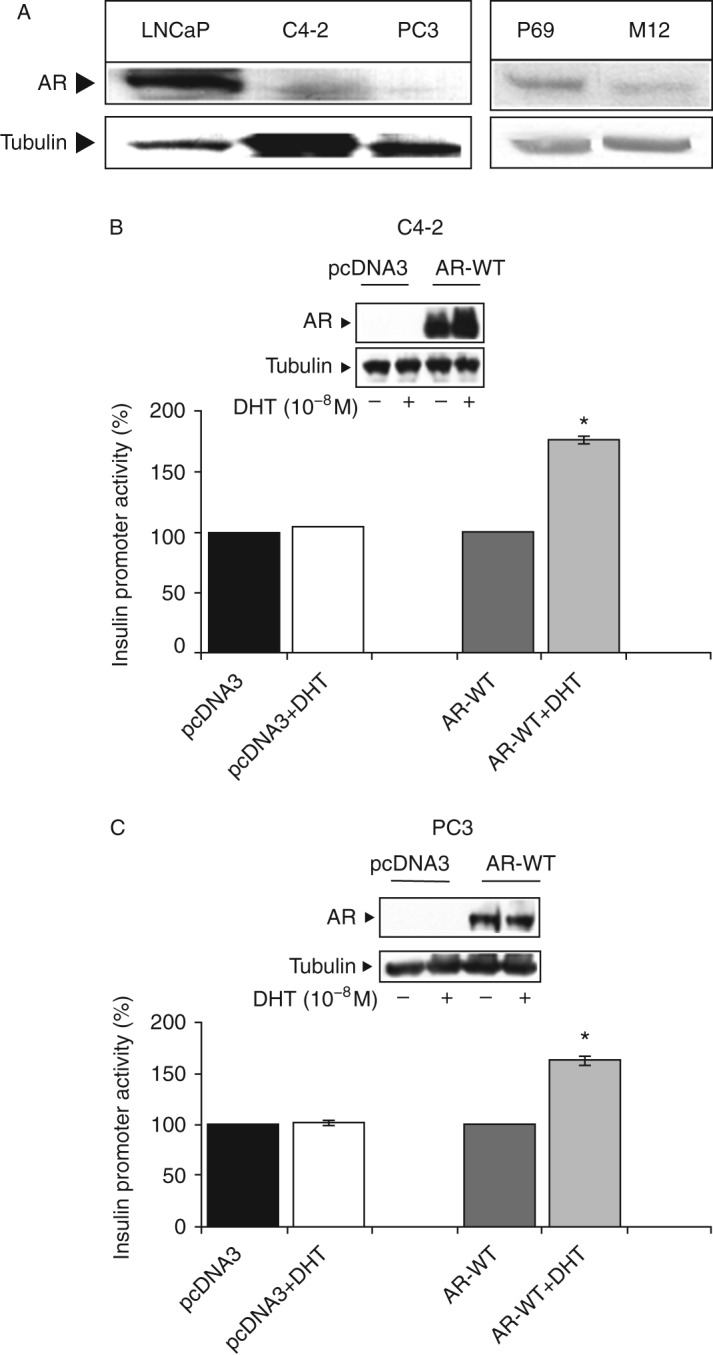
Expression of AR in prostate cancer cell lines. (A) Western blot analysis of basal AR levels. Lysates were separated by 10% SDS–PAGE and AR levels were assessed as described in the ‘Subjects and methods’ section. AR levels were measured in the same membranes shown in Fig. 1. (B and C) Effect of WT-AR on *INSR* promoter activity. C4-2 (B) and PC3 (C) cells were stably transfected with a WT-AR expression vector (WT-AR, two right bars in each panel) or an empty pcDNA3 vector for control purposes (pcDNA3, two left bars in each panel). Stable clones were transiently co-transfected with an *INSR* reporter construct and a β-galactosidase vector. After 24 h, the medium was replaced with a full medium, and the cells were incubated for an additional 24 h in the presence of 10^−8^ DHT (columns 2 and 4 in each panel) or ethanol (columns 1 and 3 in each panel). After 48 h, the cells were harvested and luciferase and β-galactosidase activities were measured. Promoter activities are expressed as luciferase levels normalized to β-galactosidase values. A value of 100% was given to the promoter activity generated by the empty reporter plasmid in the absence of ligand treatment. Bars are means±s.e.m. of three independent experiments in duplicate wells. *P<0.05 vs control cells. AR was detected by western blot analysis of C4-12 and PC3 stable cells (WT-AR or empty pcDNA3 vector), treated and untreated with DHT (insets).

The levels of endogenous IGF1R and INSR in five PCa cell lines (Fig. 1) were first evaluated. Western blot analysis revealed that the LNCaP cell line expressed relatively high INSR levels and low IGF1R levels. By contrast, the C4-2 cell line, a highly tumorigenic derivative of the LNCaP cell line, exhibited enhanced levels of both INSR and IGF1R. On the other hand, the PC3 cell line, a tumorigenic and metastatic cell line, expressed very low levels of both receptors. In addition, IGF1R and INSR levels were significantly higher in the non-tumorigenic prostate epithelial cell line P69 than in its metastatic derivative, the M12 cell line. Finally, basal AR levels were very low in four of the five cell lines analyzed, with the exception of the LNCaP cell line, which expressed high endogenous AR levels (Fig. 2A). These results replicate, in part, previously published data (16, 17, 18, 19).

### Effect of WT-AR on *INSR* promoter activity

To examine the hypothesis that AR regulates *INSR* gene expression at the level of transcription, stable C4-2 and PC3 cells transfected with a WT-AR vector (or empty pcDNA3 vector as control) were transiently transfected with an *INSR* promoter–luciferase reporter construct, along with a β-galactosidase vector. After 24 h, the cells were treated with 10^−8^ M DHT (or ethanol, as control) for an additional 24 h and, at the end of this period, were harvested and luciferase and β-galactosidase activities were measured as described previously (19). As shown in Fig. 2B and C, the expression of WT-AR (in the absence of androgen treatment) had no effect on *INSR* promoter activity. By contrast, DHT treatment enhanced *INSR* promoter activity by ∼175% in the WT-AR-expressing C4-2 cells (Fig. 2B) and by ∼165% in the WT-AR-expressing PC3 cells (Fig. 2C). DHT treatment had no effect in the control, pcDNA3-transfected cells (Fig. 2B and C).

### Activation of INSR and IGF1R by insulin and IGF1: time dependency studies

**Figure 3:**
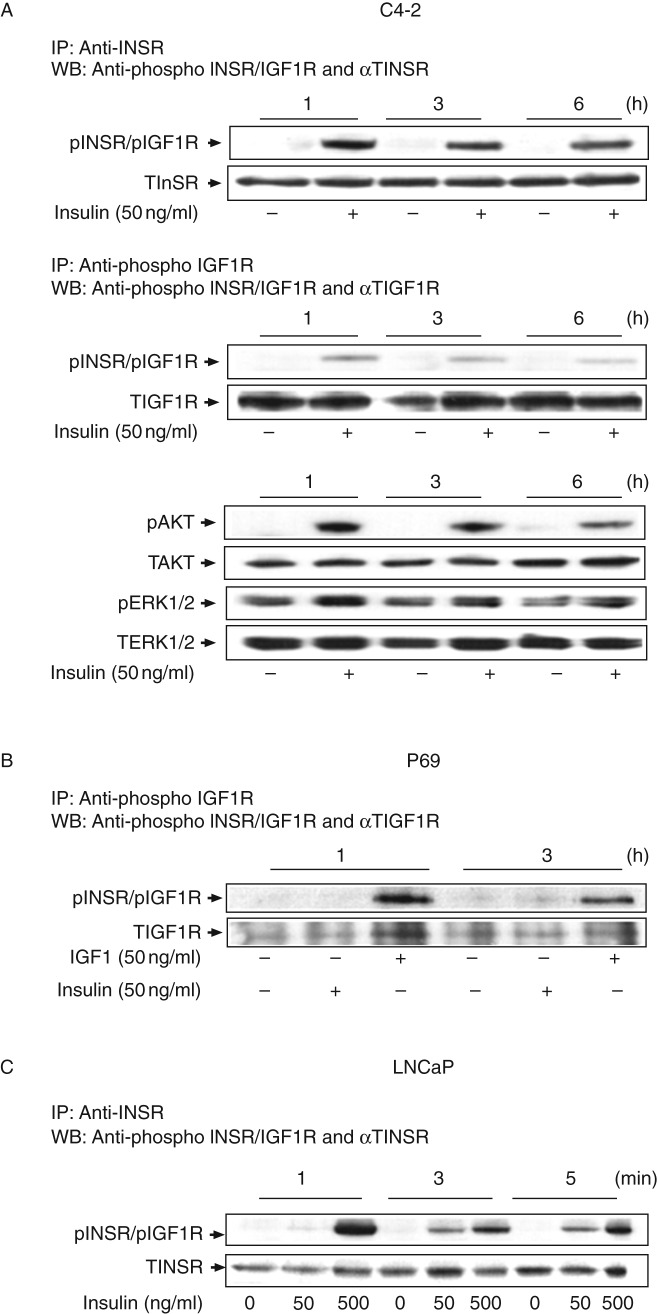
Time-dependent activation of INSR and IGF1R by insulin or IGF1 in PCa cell lines. C4-2 (A) and P69 (B) cells were treated for 1–6 h with insulin (A and B) or IGF1 (B) at a dose of 50 ng/ml, after which the cells were harvested and lysed as described in the ‘Subjects and methods’ section. Whole-cell lysates (70 μg) were immunoprecipitated with anti-INSR or anti-IGF1R, electrophoresed, and blotted with anti-phospho-INSR/IGF1R. The membranes were then incubated with anti-INSR (A) or anti-IGF1R (B) as a loading control. Autoradiographs correspond to representative experiments repeated three times with similar results. (C) Short-term stimulation of INSR by insulin in LNCaP cells. Immunoblots for pAKT, TAKT, pERK1/2, and TERK1/2 are shown in (A). Results of an illustrative experiment, repeated three times with similar results, are shown in the figure.

To identify the specific receptor(s) activated by insulin in a time-dependent manner, confluent C4-2 (Fig. 3A) and P69 (Fig. 3B) cells were maintained overnight in a starvation medium, after which they were treated with physiological doses of insulin or IGF1 (50 ng/ml) for long periods of time (1, 3, or 6 h). In addition, LNCaP cells were treated with 50–500 ng/ml of insulin for short periods of time (1, 3, or 5 min) (Fig. 3C). Cell lysates were immunoprecipitated with anti-INSR (Fig. 3A and C) or anti-IGF1R (Fig. 3A and B) for 24 h and then immunoblotted with a phospho-INSR/IGF1R antibody. The results revealed that insulin stimulated INSR phosphorylation in a time-dependent fashion over both the long-term (maximal effect at 1 h) and short-term (maximal effect at 1 min) time frames. Moreover, treatment with insulin slightly increased IGF1R phosphorylation in C4-2 cells in a time-dependent manner (Fig. 3A). No changes in total INSR or IGF1R levels were observed. Insulin also stimulated AKT and ERK1/2 phosphorylations in a time-dependent manner in C4-2 cells, with maximal phosphorylation being observed at 1 h (Fig. 3A). No changes in total AKT and ERK1/2 levels were observed. Furthermore, immunoprecipitation (IP) assays revealed that insulin was unable to activate the IGF1R in P69 cells (Fig. 3B). Together, data showed that at physiological doses of insulin or IGF1, each receptor was preferentially activated by its cognate ligand. Minimal cross-activation of the IGF1R by insulin was observed in C4-2 cells, whereas insulin was unable to stimulate it in P69 cells (Fig. 3B). Finally, only very high doses of insulin (500 ng/ml) could activate the INSR in LNCaP cells (Fig. 3C).

### Dose dependency analyses of INSR/IGF1R activation

**Figure 4:**
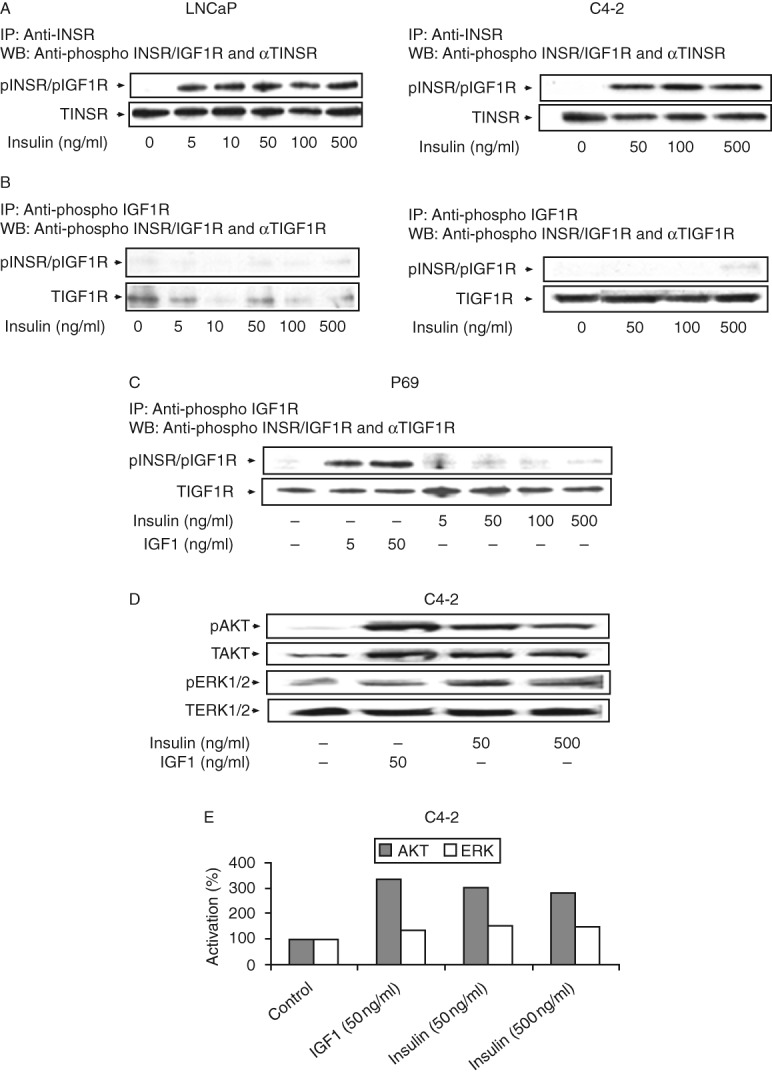
Dose-dependent activation of INSR, IGF1R, and downstream signaling proteins by insulin or IGF1. LNCaP, C4-2, and P69 cells were treated with insulin or IGF1 for 10 min at the indicated doses, after which the cells were harvested and lysed. Lysates were immunoprecipitated with anti-INSR (A) or anti-IGF1R (B and C), electrophoresed, and blotted with anti-phospho-INSR/IGF1R. The membranes were then incubated with anti-INSR (A) or anti-IGF1R (B and C) as a loading control. Lysates were analyzed for (D) phospho-AKT and total AKT and phospho-ERK1/2 and total ERK1/2. Activated AKT and ERK1/2 were measured using specific anti-phospho-AKT and anti-phospho-ERK1/2 antibodies. Autoradiographs correspond to typical experiments repeated at least three times with similar results. (E) Scanning densitometric analysis of insulin- or IGF1-stimulated AKT and ERK1/2 phosphorylations. Bars represent results of typical representative assays of three independent experiments. A value of 100% was given to the basal phosphorylation levels in untreated C4-2 cells. Increases in phospho-AKT levels were statistically significant (*P*<0.05) in comparison with untreated cells at all doses.

To identify the specific receptor(s) responsible for mediating insulin action in PCa, LNCaP, and C4-2 cells were starved overnight and then treated with increasing doses of insulin (5–500 ng/ml) for 10 min. The cells were then collected and receptor activation was assessed by IP assays, as described above. Briefly, lysates were immunoprecipitated with anti-INSR (Fig. 4A) or anti-IGF1R (Fig. 4B) for 24 h, electrophoresed through 10% SDS–PAGE, and immunoblotted with anti-phospho-INSR/IGF1R. As shown in Fig. 4A, insulin activated the INSR at all doses in both cell lines, although no dose–response activation pattern was observed. By contrast, insulin was unable to activate the IGF1R in either cell line, regardless of the IGF1R levels. Similarly, IP assays revealed that insulin (between 5 and 500 ng/ml) did not activate the IGF1R in P69 cells. In comparison, IGF1 stimulated IGF1R phosphorylation at doses as low as 5 ng/ml (Fig. 4C).

Next, the effects of IGF1 and insulin on downstream signaling pathways induced by the IGF1R and INSR were examined in C4-2 cells (Fig. 4D and E). As expected, IGF1 (50 ng/ml, 10 min) stimulated AKT (334%) and ERK1/2 (134%) phosphorylations. Similarly, insulin (50 and 500 ng/ml, 10 min) increased phospho-AKT (305% at 50 ng/ml and 284% at 500 ng/ml) and phospho-ERK1/2 (147% at 50 ng/ml and 151% at 500 ng/ml) levels.

### Evaluation of insulin-induced proliferation in Pca-derived cell lines

**Figure 5:**
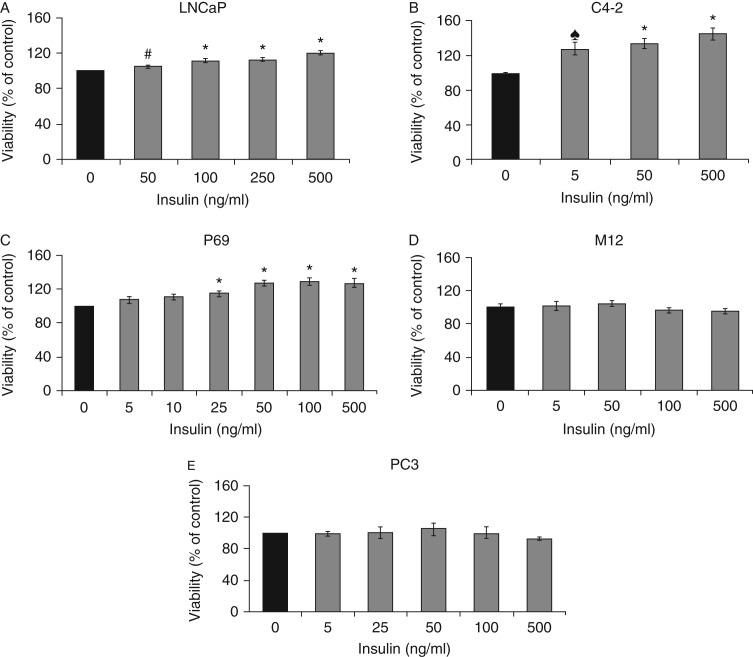
Effect of insulin on PCa cell proliferation. The cells were plated in six-well or 24-well plates at a density of 5×10^4^ cells/well for the LNCaP cell line (A), 2×10^4^ cells/well for the C4-2 cell line (B), 2×10^4^ cells/well for the P69 cell line (C), 1×10^4^ cells/well for the M12 cell line (D), and 3×10^4^ cells/well for the PC3 cell line (E). The cells were treated with the indicated insulin doses (or left untreated) for 48 h, after which proliferation rates were measured by hemocytometer or by a MTT assay. A value of 100% was assigned to the number of cells in the absence of hormone. Bars represent means±s.e.m. of three independent experiments, each carried out in triplicate samples. ^# or ♠^*P*<0.01 vs untreated cells and *P<0.05 vs untreated cells.

To evaluate the proliferative effect of insulin on PCa cells, serum-starved LNCaP, C4-2, P69, M12, and PC3 cell lines were exposed to increasing doses of insulin (5–500 ng/ml). After 48 h, proliferation was assessed by hemocytometer (P69, M12, and PC3) or by a MTT assay (LNCaP and C4-2). A dose-dependent stimulatory effect was observed in LNCaP, C4-2, and P69 cells at all insulin doses, although the insulin-induced proliferation rates varied between the cell lines. In LNCaP cells, insulin at a dose of 50 ng/ml induced 5±0.8% stimulation compared with untreated cells (Fig. 5A). Higher insulin doses (100, 250, and 500 ng/ml) stimulated proliferation by 12±1.6, 13±1.1, and 20±2.6% respectively. C4-2 cells exhibited a greater sensitivity to insulin. At the lowest dose tested (5 ng/ml), insulin induced 28±7% stimulation compared with control cells (*P*<0.05; Fig. 5B). Higher insulin doses (50 and 500 ng/ml) induced proliferation by 34±5.6 and 45±6.7% respectively. In P69 cells, insulin doses higher than 25 ng/ml led to significant increases in proliferation rates, whereas doses of 5–10 ng/ml had no effect (Fig. 5C). Insulin had no mitogenic effect on M12 or on PC3 cells at any concentration (Fig. 5D and E).

### Effect of insulin on cell-cycle dynamics in PCa cell lines

The effect of insulin on cell-cycle progression was examined in LNCaP, C4-2, and PC3 cells using fluorescence activated cell sorting (FACS) analysis (Table 1). In LNCaP cells, low-to-medium doses of insulin (5 and 50 ng/ml) had no effect on cell-cycle progression compared with control cells. However, higher insulin doses (500 ng/ml) stimulated cell-cycle progression, as reflected by the low proportion of cells in the G0/G1 phase (46.9±1.7%) and the high proportion in the S+G2/M phase (53.1±1.1%) compared with untreated cells (57.8±2.7% in G0/G1 and 42.2±1.3% in S+G2/M). In C4-2 cells, insulin stimulated cell-cycle progression at doses of 50 ng/ml (55.8±2.4% in S+G2/M) or 500 ng/ml (58.4±1.5% in S+G2/M), but not at 5 ng/ml. Finally, insulin had no effect on cell-cycle progression in PC3 cells. In summary, cell-cycle analysis revealed that insulin increased the proportion of cells in the S+G2/M phase and caused a decrease in the proportion of cells in the G0/G1 phase by ∼19% in the LNCaP cell line and by about 9–14.4% in the C4-2 cell line.

### Compensatory increase in INSR signaling following IGF1R blockade

**Figure 6:**
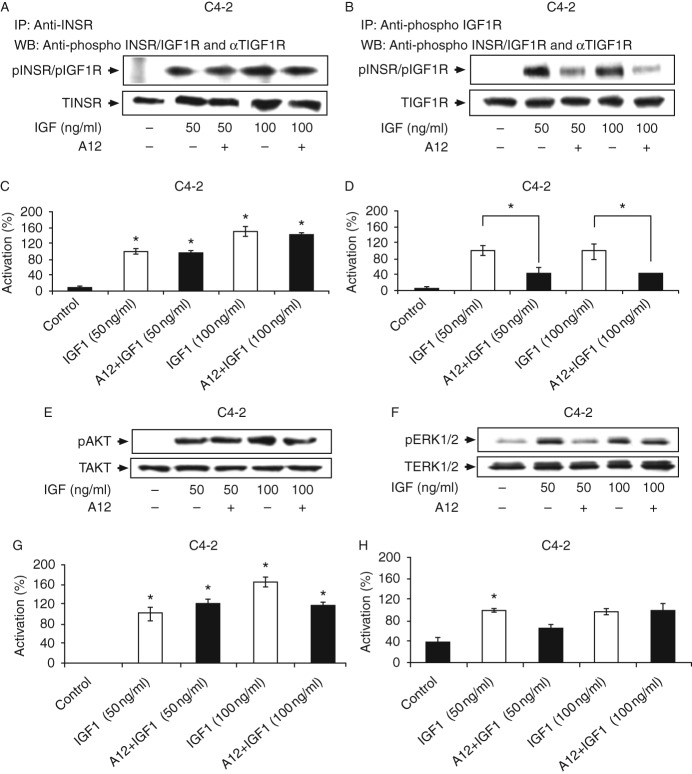
Effect of cixutumumab (A12) on the IGF1-stimulated INSR, IGF1R, AKT, and ERK phosphorylations in C4-2 cells. (A and B) Effect of A12 on the IGF1-induced INSR and IGF1R phosphorylations. C4-2 cells were treated with A12 for 24 h, in the presence of IGF1 (50 or 100 ng/ml) during the last 10 min of incubation. At the end of the incubation period, the cells were lysed and immunoprecipitated with INSR (A) or IGF1R (B) antibodies, electrophoresed through SDS–PAGE, and immunoblotted with anti-phospho-IGF1R/INSR, anti-total INSR, or anti-total IGF1R. (C and D) Scanning densitometric analysis of phospho-INSR and phospho-IGF1R. Optical density is expressed as phospho-INSR (C) or phospho-IGF1R (D) values normalized to the corresponding total INSR or IGF1R levels. Bars represent means±s.e.m. of three independent experiments. *P<0.05 vs untreated cells (C) and *P<0.05 vs A12+IGF1-treated cells (D). (E and F) Effect of A12 on the IGF1-induced AKT and ERK1/2 phosphorylations. C4-2 cells were processed as described above, lysed, electrophoresed, and immunoblotted with anti-phospho-AKT (E) or phospho-ERK1/2 (F) and total AKT or total ERK1/2. (G and H) Scanning densitometric analysis of phospho-AKT and phospho-ERK1/2. Optical density is expressed as phospho-AKT (G) or phospho-ERK1/2 (H) values normalized to the corresponding total AKT or ERK1/2 levels. Bars represent means±s.e.m. of three independent experiments. *P<0.05 vs control cells (G) and *P<0.05 vs A12+IGF1-treated cells (H). Results of typical experiments, repeated three times with similar results, are shown in the figure.

**Figure 7:**
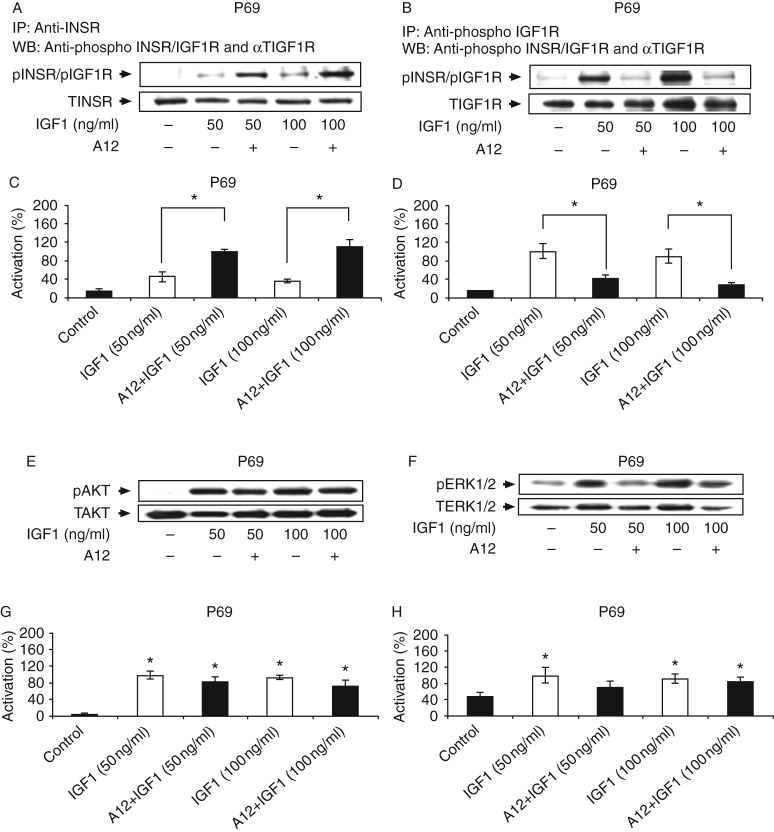
Effect of cixutumumab (A12) on the IGF1-stimulated INSR, IGF1R, AKT, and ERK phosphorylations in P69 cells. P69 cells were incubated with the A12 antibody in the presence or absence of IGF1. Levels of phosphorylated and total proteins were measured by western blot analysis. For phospho-receptor measurement, cell extracts were immunoprecipitated with anti-INSR (A) or IGF1R (B), electrophoresed, and immunoblotted with anti-phospho-INSR/IGF1R. AKT (E) and ERK1/2 (F) phosphorylation levels were measured using specific anti-phospho antibodies. Results of a typical experiment, repeated three times with similar results, are shown in the figure. Scanning densitometric analysis of the effect of A12 on INSR (C), IGF1R (D), AKT (G), and ERK1/2 (H) activation. Bars represent phospho-protein values normalized to the corresponding total protein levels. Bars are means±s.e.m. of three independent experiments. *P<0.05 vs control cells (G and H) and *P<0.05 vs A12+IGF1-treated cells (C and D).

Next, we evaluated whether IGF1R blockade would lead to IGF1 activation of the INSR in C4-2 and P69 cells. These cells were chosen because they express similar levels of both receptors (Fig. 1). The selective IGF1R antibody cixutumumab (A12), a fully human monoclonal IgG1, was used in these experiments. A12 has been shown to bind with high affinity to the IGF1R, but not to the INSR, and to inhibit ligand-dependent receptor activation and downstream signaling (20). Starved cells were incubated with IGF1 (50–100 ng/ml) for 10 min in the presence or absence of A12, and IGF1R and INSR phosphorylation levels were measured by IP followed by immunoblotting. Lysates were immunoprecipitated with anti-INSR (Figs 6A and 7A) or anti-IGF1R (Figs 6B and 7B) for 24 h and immunoblotted with anti-phospho-INSR/IGF1R. As expected, IGF1 strongly activated its cognate receptor in both cell lines (Figs 6B and 7B) while also leading to the cross-activation of the INSR (Figs 6A and 7A). In C4-2 cells, A12 treatment attenuated the IGF1-induced IGF1R phosphorylation at both doses of the ligand (Fig. 6B and D), but had no effect on the IGF1-induced INSR activation (Fig. 6A and C). Similarly, A12 abolished the IGF1-induced IGF1R phosphorylation in P69 cells at both ligand concentrations (Fig. 7B and D). Interestingly, in these cells, A12 treatment enhanced the IGF1-stimulated INSR activation (55 and 64% increases at 50 and 100 ng/ml of IGF1 respectively; Fig. 7A and C).

Western blot analysis of downstream AKT and ERK mediators revealed that A12 reduced the IGF1-induced AKT phosphorylation by 40% in C4-2 cells. The inhibitory effect of the antibody was observed only at the high ligand dose (100 ng/ml; Fig. 6E and G). On the other hand, A12 inhibited the IGF1-induced ERK1/2 activation by 34% at the low ligand dose (50 ng/ml), but had no effect at the high dose (Fig. 6F and H). In P69 cells, A12 had no inhibitory effect on the IGF1-stimulated AKT activation (Fig. 7E and G), although it reduced the IGF1 (50 ng/ml)-induced ERK1/2 phosphorylation by 30% (Fig. 7F and H). Collectively, these data indicate that i) IGF1 activates IGF1R and INSR in both C4-2 and P69 cell lines; ii) INSR activation by IGF1 is not affected by A12 treatment (in comparison with the complete inhibition of IGF1R); and iii) downstream signaling cascades are inhibited by A12 only to a limited extent. These findings suggest that the INSR pathway can compensate for IGF1R inhibition by A12 with an increase in IGF1-stimulated INSR activation.

### Mitogenic effect of IGF1 following IGF1R inhibition

**Figure 8:**
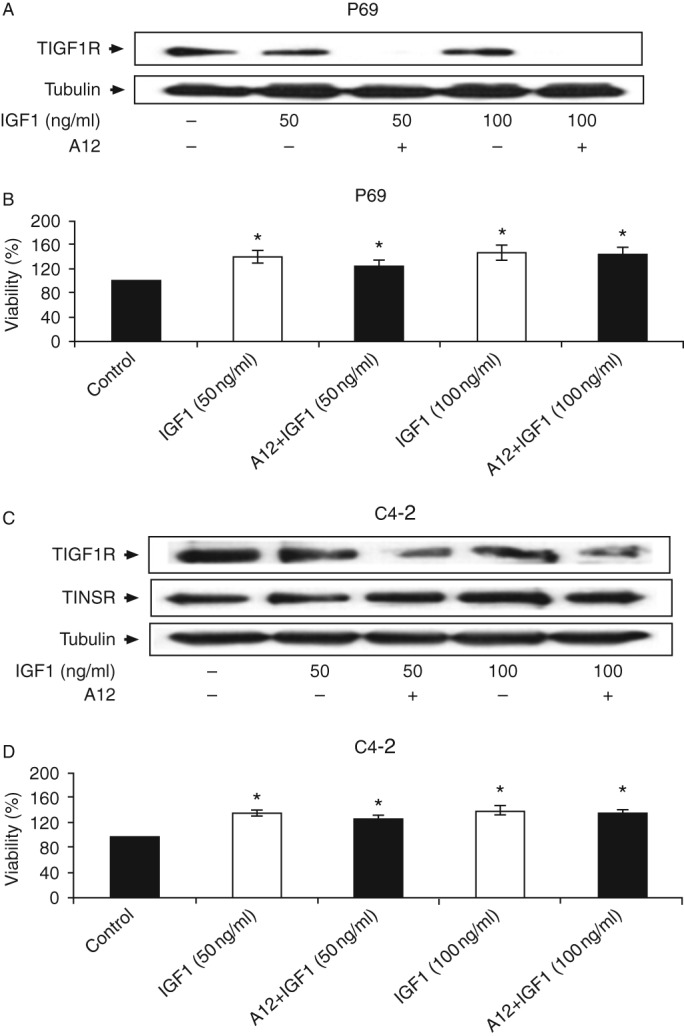
Effect of A12 on IGF1R levels. P69 and C4-2 cells were treated with the A12 antibody for 48 h in the presence or absence of IGF1. IGF1R levels were measured by western blot analysis (A and C). Effect of A12 on P69 and C4-2 cell proliferation. The cells were plated in 24-well plates at a density of 2×10^4^ cells/well for the P69 cell line (B) and 2×10^4^ cells/well for the C4-2 cell line (D). The cells were treated with IGF1 or IGF1 in combination with A12 for 48 h and the proliferation rates were measured by a MTT assay. The number of cells at time 0 was assigned a value of 100%. Bars represent means±s.e.m. of three independent experiments, each carried out in triplicate. *P≤0.05 vs control cells.

 To evaluate the ability of IGF1 to elicit mitogenic activities following IGF1R blockade, P69 cells were treated with A12 in the presence or absence of IGF1 (50 and 100 ng/ml) for 48 h. At the end of the incubation period, IGF1R expression and cell viability were examined. The results of western blot analysis indicated a drastic reduction in IGF1R expression (Fig. 8A). Despite the decrease in IGF1R levels, the capacity of IGF1 to stimulate proliferation was not affected (Fig. 8B). Hence, IGF1 (50 and 100 ng/ml) enhanced proliferation by 24–46% both in the absence and in the presence of A12. Similar results were obtained for C4-2 cells (Fig. 8C and D). Furthermore, similar results were obtained using tyrphostin AG1024, a specific IGF1R tyrosine kinase inhibitor (data not shown). In summary, our results indicate that INSR compensated for and mediated IGF1 mitogenic signals after IGF1R inhibition.

## Discussion

Epidemiological studies suggest an association between high insulin levels and PCa risk (21). In addition, increased INSR levels have been measured in cells derived from primary PCa tumors (22). The fact that hyperinsulinemia is a pathological hallmark of type 2 diabetes, along with the structural and functional homology between INSR and IGF1R, prompted us to further investigate the potential roles of insulin and INSR in PCa development and progression. The ability of insulin and IGF1 to activate the opposite receptor is documented in the literature, reporting that in some cases IGF1R can mediate metabolic activities (10), whereas INSR can be involved in growth, anti-apoptotic, and developmental activities (23). However, no prospective studies have directly assessed how the activation of INSR or IGF1R leads to divergent biological events. In this respect, it is legitimate to question whether the distinct biological effects elicited by the activated receptors can be explained only by differential ligand affinity, divergent tissue distribution, differences in the internalization of the receptors, or structural differences in the β-subunit, specifically in the C-terminus (24).

The role of the insulin/IGF signaling pathways in PCa has been a topic of major interest in recent years. In this study, we investigated whether insulin can directly induce mitogenic activity in non-tumorigenic, tumorigenic, and metastatic PCa cell lines and evaluated whether this insulin activity is mediated via its cognate receptor. Our results indicated that insulin activated the INSR, but not the IGF1R in PCa cells (even at high doses). IGF1, on the other hand, activated both receptors. In addition, insulin activated the downstream signaling pathways (PI3K/AKT and Ras-Raf-MEK/ERK) in C4-2 cells.

Insulin exhibited a mitogenic activity in the LNCaP, C4-2, and P69 PCa cell lines. However, the magnitude of this effect differed among the various cell lines. Moreover, insulin at high doses stimulated cell-cycle progression in LNCaP and C4-2 cells. By contrast, insulin did not elicit any mitogenic activity in PC3 and M12 cells at any dose. Of importance is that variations in the rates of insulin secretion have been shown to influence cancer risk and prognosis among individual patients (21). In contrast to our results, Heidegger *et al*. (3) showed that PC3 and LNCaP cells express both IGF1R and INSR and that IGF1 or insulin separately and in combination enhances the proliferation of these cells. Differences among studies could be explained by the use of different cell clones or different experimental conditions. Both cell types have been employed as models for PCa in many studies and, for the most part, the expression patterns of both receptors are similar to that shown in Fig. 1 (18, 25, 26). Mitogenic signaling by INSR has been described in some tumor models and examples have been provided in which the IGF1R or INSR compensates for the inhibition of the opposite receptor. In fact, the activation of INSR by IGF2 was first described in a mouse development model. In this model, IGF2-activated INSR was shown to compensate for IGF1R interruption to rescue embryonic growth (27). Additional studies have demonstrated intensified insulin signaling when IGF1R is interrupted in tumor cells (28). Hence, our results are consistent with the concept that insulin has the ability to exhibit mitogenic activity in PCa. The activation pattern of INSR suggests that it is directly involved in the mediation of this mitogenic activity and, therefore, might be relevant in translational terms for the development of INSR inhibitors. Finally, the potential involvement of hybrid receptors composed of an INSR hemireceptor linked to an IGF1R hemireceptor in the mediation of insulin/IGF1 action in PCa cells cannot be excluded.

Of interest is that we provide herein evidence of the transcriptional regulation of the *INSR* promoter by the androgen-stimulated AR in C4-2 and PC3 cells. Little information is available regarding the joint regulation of the insulin/IGF1 and androgen signaling pathways in PCa. Changes in AR structure and expression are responsible for the progression of androgen-dependent tumors to a more aggressive, hormone-refractory, androgen-independent forms. The progression of tumors from an organ-confined, androgen-sensitive disease to a metastatic one is associated with the deregulation of AR-regulated targets and upregulation of AR expression (29). We have previously shown that WT, but not mutant, AR along with DHT treatment increases *IGF1R* promoter activity and endogenous IGF1R levels (18). In addition, we provided evidence that the activated WT-AR enhances *IGF1R* transcription via a mechanism that involves AR binding to the *IGF1R* promoter and *AR* mutations alter the ability of the mutated protein to regulate IGF1R expression. The mechanisms responsible for the AR activation of INSR need to be explored further.

The IGF1R has been identified as an attractive therapeutic target in oncology. Several strategies for IGF1R inhibition have been developed, including antibodies against the IGF1/IGF2 ligands, IGF1R-blocking antibodies, and IGF1R tyrosine kinase domain inhibitors. In addition to the challenges associated with the development of most inhibitors, including specific tumor delivery and dose-related issues, the large homology that exists between this receptor and the related INSR should be taken into account while developing IGF1R inhibitors. IGF1R-targeting therapies have been reported to cause various side effects, most commonly hyperglycemia, a hallmark of type 2 diabetes (3). Antibody A12 has been shown to specifically inhibit the IGF1 activation of IGF1R, but not of INSR, and to downregulate IGF1R, but not INSR, expression levels in PCa cells. Despite significant downregulation of IGF1R after A12 treatment, IGF1 treatment increased cell viability. Taken together, our results confirm that INSR expression might constitute a compensatory mechanism following specific IGF1R inhibition in PCa. This mechanism might lead to the mitogenic actions of IGF1 via the INSR. This compensation loop emphasizes the need to develop new therapeutics to co-target IGF1R and INSR in PCa. Finally, further research is needed to examine additional mitogenic activities induced by insulin relevant to PCa development and progression, and to explore the molecular mechanism of INSR expression and activity.

## Figures and Tables

**Table 1:** Effect of insulin on the cell cycle in PCa cell lines. LNCaP, C4-2, and PC3 cells were seeded in quadruplicate dishes, serum-starved for 24 h, and incubated with insulin (5–500 ng/ml) for 48 h (or left untreated, controls). Cell-cycle progression was assessed by FACS analysis. The results represent the values of a typical experiment, repeated three times.

**Insulin** (ng/ml)	**G0/G1**	**S+G_2_/M**
LNCaP		
0	57.8±2.7%	42.2±1.3%
5	55.6±4.8%	44.4±2.1%
50	53.5±0.59%	46.5±3.4%
500	46.9±1.7%	53.1±1.1%^*^
C4-2		
0	48.6±1.2%	51.4±1.4%
5	47.7±0.4%	52.3±2.1%
50	44.2±0.7%	55.8±2.4%^*^
500	41.6±1.7%^*^	58.4±1.5%^*^
PC3		
0	45.24±0.5%	54.8±6.1%
5	46.27±0.8%	53.8±3.5%
50	46.05±0.4%	54.03±0.4%
500	45.65±3%*	54.7±9.2%^*^

* Significantly different vs control untreated cells (*P*<0.05).
